# Editorial: Insights in biosafety and biosecurity 2022: Novel developments, current challenges, and future perspectives

**DOI:** 10.3389/fbioe.2022.1118506

**Published:** 2023-01-12

**Authors:** Segaran P. Pillai, Alan Raybould

**Affiliations:** ^1^ Office of the Commissioner, Food and Drug Administration, Department of Health and Human Services, Silver Spring, MD, United States; ^2^ Innogen Institute, Science Technology and Innovation Studies Department, University of Edinburgh, Edinburgh, United Kingdom

**Keywords:** biosafety, biosecurity, novel developments, current challenges, future perspective

Biosafety and Biosecurity covers the relationship between bioengineering and biotechnology and the assessment and management of risk to human and animal health and the environment. This relationship includes both the risk posed by research and its application and the research and application to reduce risk through bioengineering and biotechnology. To this end, Frontiers developed a Research Topic entitled “*Insights in Biosafety and Biosecurity 2021: Novel Developments, Current Challenges, and Future Perspectives*” that encompasses human health and environmental risk assessments of bioengineering and biotechnology when used as a tool in basic or applied research.

In 2021, 9 manuscripts were originally submitted for this Research Topic. Of those 8 (89%) were accepted. Bouattour et al. reviewed recent progress in the field of “brain models stimulating geometrical, physical and biochemical properties of the human brain” addressing the growing body of evidence for the need of brain surrogates for researchers and physicians in the medical field. Currently these models are being used for education and training or to verify the appropriate functionality of medical devices, and more recently to assess the biocompatibility of implantable devices that lack an understanding of whether components leach from such devices. The authors also suggested avenues of research for the analysis of leached materials from implantable devices.

Mbaya et al. discussed their concerns that “regulatory frameworks can facilitate or hinder the potential of genome editing to contribute to sustainable agricultural development” because the advent of new breeding techniques, in particular genome editing, has provided more accurate and precise ways to introduce targeted changes in the genome of both plants and animals. Regulatory frameworks can affect different stakeholders who have different applications such as comparison of transgenics. Although countries are reviewing applications involving organisms that were subjected to genome editing, the authors suggest that early stakeholder engagement and communication(s) with the public be emphasized to foster public acceptance even before products are ready for market. Furthermore, global cooperation and consensus on Research Topic cutting across regions will be crucial in avoiding regulatory-related bottlenecks that affect global trade and agriculture.

Willow et al. discussed “uniting RNA interference (RNAi) technology and conservation biocontrol to promote global food security and agrobiodiversity” to address habitat loss and fragmentation, and to study the effects of pesticides that contribute to biodiversity losses and unsustainable food production. The authors advocate for combining conservation biocontrol-enhancing practices with the use of RNAi pesticide technology, which demonstrates remarkable target specificity *via* double stranded RNA’s sequence-specific mode of action. They also shared the importance of interdisciplinary studies to bring together biocontrol-enhancing techniques and RNAi for various pest systems to address this global problem.

Xue et al. discussed ‘‘*towards better governance on biosafety and biosecurity: China’s advances and perspectives in medical biotechnology legislation*.” They systematically analyzed 89 laws, rules, measures, guidelines and views from 1985 to 2022 and determined that 28 were involved in medical biotechnology legislation, including the recently passed Biosecurity Law.

They also shared the advances China is making in this area by incorporating biotechnology-related biosafety and biosecurity into their national strategic goals and the importance of “top-down” formulation of general objectives by the active political leadership and “bottom-up” innovation in the implementation as the keys to achieving these goals.

Many countries have worked diligently to establish and implement policies and processes such as the Select Agents and Toxins regulation, for the accountability and control of high consequence pathogens and toxins that can have a significant public health impact if misused. Pillai et al. described a robust process using Multi-Criteria Decision Analysis and Decision Support Framework logic tree approaches for evaluating high consequence human pathogens to support their inclusion or exclusion on a select agents list for oversight and control. In another contribution, Pillai et al. also developed and described a process for evaluating high consequence toxins for selection and establishment of exclusion limits using a Decision Support Framework logic tree approach coupled with scenario-based modeling studies to support the Federal Select Agent Program. This is a critical path forward for these regulations; to be able to identify agents and toxins that no longer pose significant risk can lead to their delisting when the list is reviewed biannually. The authors hope that as others learn about and utilize these new approaches, they can have a larger impact by supporting global partners in their select agents and toxins determinations.

A laboratory quality management system (LQMS) is an essential element for the effective and safe operation of laboratories conducting research, clinical testing, or production/manufacturing activities. As technology continues to rapidly advance and new challenges arise, laboratories worldwide have responded with innovation and process changes to meet the continued demand. Pillai et al. shared that quality in the laboratory is a multi-faceted system that involves much more than ensuring the continued reliability and repeatability of experiments and tests, accuracy of data, and safety of personnel. Different laboratories conduct very different types of work, such as basic research, clinical testing/diagnostics, regulatory testing, etc.; however, there is currently not a one-size-fits-all approach that can be effectively used by all types of laboratories when it comes to the implementation and adoption of a robust LQMS. In this paper, the authors compared the different ISO standards to the 12 Quality Systems Essentials identified in the WHO LQMS Handbook while taking into consideration the different types of laboratory work to provide guidance on how to establish a robust quality management program that best meets the laboratory’s needs.

Nanotechnology is a revolutionary science involving fabricating and dealing with nanometer particles (NPs) comprised of various materials. Nanotechnology has endowed us with a new robust platform with a wide range of potential and practical applications including medicine, diagnostic devices, agriculture, catalysts, cosmetics, biological sensors, etc. Zhang et al. discussed the unique physicochemical properties that metal-based NPs confer, not only their promising biological effects but also the unexpected toxic threats they pose to the human body. They also summarized our current knowledge about metal-based NPs, including the physicochemical properties affecting their toxicity, mechanisms of toxicity, their toxicological assessment, the potential strategies to mitigate their toxicity and status of regulatory controls.

To support the safety and security mission it is critical to ensure the protection of workers and prevent work-related injuries, illnesses, and deaths by setting and enforcing standards, and by providing training, outreach, education, and assistance. It is also important to establish policies and practices to ensure that the work is executed safely by employing various controls (engineering and administrative), personal protective equipment, leveraging new biotechnology, performing appropriate hazards identification, risk assessments and implementing risk mitigation strategies.

In addition to the contributions of these authors in support of biosafety and biosecurity, scientists from around the world are working to enhance our knowledge, understanding, develop better and safer tools, technologies, engineering and administrative controls, personal protective equipment and share lessons learned and best practices to ensure the safe conduct of laboratory science and the safety and health of our community. The editors of this Research Topic would like to thank the many authors for their contributions to enhance our knowledge in the area of biosafety and biosecurity and their dedication to public health.

